# A Review of the Interactions between Wheat and Wheat Pathogens: *Zymoseptoria tritici*, *Fusarium* spp. and *Parastagonospora nodorum*

**DOI:** 10.3390/ijms19041138

**Published:** 2018-04-10

**Authors:** Adrian Duba, Klaudia Goriewa-Duba, Urszula Wachowska

**Affiliations:** 1 Department of Entomology, Phytopathology and Molecular Diagnostics, University of Warmia and Mazury in Olsztyn, Prawocheńskiego 17, 10-719 Olsztyn, Poland; adrian.duba@uwm.edu.pl (A.D.); urszula.wachowska@uwm.edu.pl (U.W.); 2 Department of Plant Breeding and Seed Production, University of Warmia and Mazury in Olsztyn, pl. Łódzki 3, 10-724 Olsztyn, Poland

**Keywords:** *Zymoseptoria tritici*, *Parastagonospora nodorum*, *Fusarium*, anatomical barriers, pattern-triggered immunity

## Abstract

*Zymoseptoria tritici* is a hemibiotrophic pathogen which causes Septoria leaf blotch in wheat. The pathogenesis of the disease consists of a biotrophic phase and a necrotrophic phase. The pathogen infects the host plant by suppressing its immune response in the first stage of infection. Hemibiotrophic pathogens of the genus *Fusarium* cause Fusarium head blight, and the necrotrophic *Parastagonospora* *nodorum* is responsible for Septoria nodorum blotch in wheat. Cell wall-degrading enzymes in plants promote infections by necrotrophic and hemibiotrophic pathogens, and trichothecenes, secondary fungal metabolites, facilitate infections caused by fungi of the genus *Fusarium*. There are no sources of complete resistance to the above pathogens in wheat. Defense mechanisms in wheat are controlled by many genes encoding resistance traits. In the wheat genome, the characteristic features of loci responsible for resistance to pathogenic infections indicate that at least several dozen genes encode resistance to pathogens. The molecular interactions between wheat and *Z*. *tritici*, *P*. *nodorum* and *Fusarium* spp. pathogens have been insufficiently investigated. Most studies focus on the mechanisms by which the hemibiotrophic *Z*. *tritici* suppresses immune responses in plants and the role of mycotoxins and effector proteins in infections caused by *P*. *nodorum* and *Fusarium* spp. fungi. Trichothecene glycosylation and effector proteins, which are involved in defense responses in wheat, have been described at the molecular level. Recent advances in molecular biology have produced interesting findings which should be further elucidated in studies of molecular interactions between wheat and fungal pathogens. The Clustered Regularly-Interspaced Short Palindromic Repeats/ CRISPR associated (CRISPR/Cas) system can be used to introduce targeted mutations into the wheat genome and confer resistance to selected fungal diseases. Host-induced gene silencing and spray-induced gene silencing are also useful tools for analyzing wheat–pathogens interactions which can be used to develop new strategies for controlling fungal diseases.

## 1. Introduction

Various species of the genus *Triticum* L., in particular the allohexaploid wheat species of the genus *T*. *aestivum* L., are among the major groups of domesticated crops [1]. In 2016, annual wheat production (mostly common wheat) reached 758.1 million tons, and it accounted for a third of the global cereal output [2]. In Poland, annual wheat production was estimated at 10.5 million tons in 2016 [3]. According to research, the gene pool of *T. aestivum* continues to decrease [4], mainly due to intensive breeding. The observed decrease in genetic diversity lowers wheat’s resistance to biotic stress. Septoria leaf blotch caused by the *Zymoseptoria tritici* (Desm.) Quaedvlieg and Crous (synonym: *Septoria tritici*), a hemibiotrophic pathogen (teleomorph *Mycosphaerella graminicola*), is able to decrease wheat yields by up to 30–50% [5]. All winter wheat cultivars are susceptible to infections caused by *Z. tritici* which is increasingly resistant to quinone outside inhibitors (QoI) and demethylation inhibitors (DMI) [6]. Hemibiotrophic pathogens of the genus *Fusarium* infect wheat between the seedling stage and the fully ripe stage. Fungal pathogens produce mycotoxins which contaminate grain and facilitate the spread of infection [7]. *Parastagonospora* (anamorph *Stagonospora* and teleomorph *Phaeosphaeria*) *nodorum* (Berk.) Quaedvlieg, Verkley and Crous, is a necrotrophic fungus which causes Septoria nodorum blotch in wheat and decreases yields by up to 20–50% [8].

At least two fungicide treatments are applied, including in the UK [9], USA [10,11], Germany [12] and Poland [13], during the growing season to protect wheat against pathogens. However, the efficiency of fungicide treatments against *Fusarium* is only 15–30% [14]. Fungicide applications increase wheat production costs and contribute to environmental pollution. Breeding wheat varieties resistant to pathogens is the most sustainable solution which increases yields and lowers production costs. Wheat grain is a staple food in the human diet, and the seeds of resistant and high-performing wheat cultivars are in high demand. In wheat breeding programs, there is a demand for new resistance genes against wheat pathogens. In 2016, the following wheat cultivars were recommended for cultivation in Poland: KWS Ozon, Fidelius, Patras, Arkadia and Artist. Of these, Arkadia was the only cultivar characterized by high resistance to *Z*. *tritici* [15].

In plants, resistance to pathogens is conditioned by the presence of constitutive barriers which include a thick cuticle layer and a waxy epidermal cuticle on aerial plant parts. The apoplast is the second potential barrier to the filtering and processing of environmental signals and transmitting them to the symplast [16]. Pathogens produce signaling molecules encoded by *avr* genes, which are recognized by plant receptors encoded by R-genes. When the signal is recognized, the plant initiates a series of defense mechanisms, including the accumulation of reactive oxygen species (ROS), changes in the conformation of proline-rich cell wall proteins, and the production of salicylic acid to induce the expression of genes encoding pathogenesis-related (PR) proteins. In all plants, resistance is conditioned by molecular interactions with pathogens. This article reviews recent trends in research into molecular interactions between wheat and the most dangerous wheat pathogens: *Z*. *tritici*, *F*. *graminearum* and *P*. *nodorum*.

## 2. The Infection Cycle of the Hemibiotrophic Fungi *Zymoseptoria tritici* and *Fusarium* spp. and the Necrotrophic Fungus *Parastagonospora nodorum*

**Figure 1:**
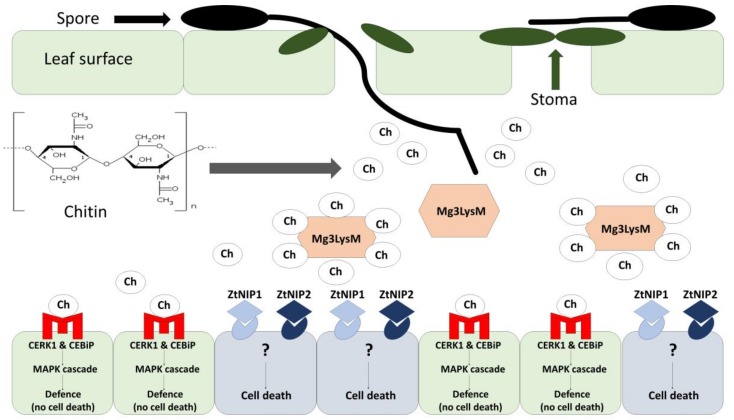
Metabolic pathway in wheat tissues infected with *Z. tritici* (adapted from Kettles and Kanyuka (2016) [19]). Ch-chitin, CEBiP-chitin elicitor-binding protein, CERK1-chitin elicitor response kinase 1, LysM-lysine motif, MAPK-mitogen-activated protein kinase, NIP-necrosis-inducing proteins.

*Zymoseptoria tritici* infects the host plant almost exclusively through leaf stomata, however less recent data suggest that *Z. tritici* is able to penetrate wheat leaves at the junctions of epidermal cells [17]. *Z. tritici* differs from other phytopathogenic fungi in that it does not produce specialized active structures during the infection and is confined to the apoplastic space of the host plant [18]. The discussed pathogen is a hemibiotroph whose life cycle consists of two distinct phases: a biotrophic phase and a necrotrophic phase. There is no evidence to indicate that specialized nutrient-absorbing structures, such as haustoria, are formed in the biotrophic phase [19,20]. Rohel et al. (2001) [20] found that *Z. tritici* takes up nutrients such as soluble carbohydrates accumulated in the apoplast. A recent analysis of changes in the transcriptome and metabolome of susceptible wheat cultivars infected by *Z*. *tritici* revealed that the pathogen’s own lipids and fatty acids are the main source of energy during the biotrophic phase. The expression of genes encoding cutinase and lipase proteins increases in the biotrophic phase, which suggests that *Z. tritici* also utilizes the nutritional resources of the host plant [21]. The first phase of *Z*. *tritici* infection is often asymptomatic because the fungus is capable or suppressing or avoiding the plant’s defense mechanisms. These processes play a key role during the infection. *Z. tritici* genes encode three proteins with lysine motifs (LysM): Mg1LysM, Mg3LysM and MgxLysM [19] (Figure 1, Table 1). Marshall et al. (2011) [22] demonstrated that the transcriptional activity of the first two motifs increases in the asymptomatic phase of *Z*. *tritici* infection, and that both motifs are capable of binding chitin, the elicitor of defense responses in plants. The Mg3LysM motif plays a key role in the interactions between wheat and *Z*. *tritici* because it is the only chitin-binding LysM that suppresses the host’s immune response in the first stage of infection [22]. Figure 1 (adapted from Kettles and Kanyuka (2016) [19]) presents the metabolic pathway in wheat tissues during *Z. tritici* infection. When pathogenic chitin is recognized, mitogens activate kinases, which initiates a defense response without cell death. 

Modifications: receptor shape, emphasis on the Mg3LysM molecular mechanism of action and the recognition of chitin fragments in *Z. tritici* mediated by CERK1 and CEBiP. In the revised manuscript, the involvement of selected molecules and genes has been disregarded in Figure 1 because their mechanisms of action are unknown. 

It appears that significant differences in the described pathogens’ infection strategy are observed mainly in the first stage of the infection process. The hemibiotrophic pathogen *Z. tritici* produces compounds that inhibit defense responses in wheat. The suppression of wheat defense responses by hemibiotrophic pathogens of the genus *Fusarium* has not been described to date. The effectors that down-regulate wheat defense responses have not been identified for *P. nodorum* either. *Fusarium* pathogens are characterized by a unique infection process which involves the production mycotoxins that increase the pathogen’s virulence. *P. nodorum* is unique in that it produces a wide range of necrotrophic effectors (toxins) that confer different levels of susceptibility across wheat cultivars. One of the best known toxins is ToxA. *P. nodorum* produces effector proteins in the first stage of infection and induces tissue necrosis in wheat. However, toxin production by *Z. tritici* has not been reported. The infection process of the described wheat pathogens is similar towards the end of tissue colonization and penetration because it relies mainly on the production of cell wall-degrading enzymes.

Under field conditions, the biotrophic (asymptomatic) stage of *Z. tritici* lasts 6–36 days. The length of this stage is determined by a combination of factors, including wheat species, pathogenic strain and weather conditions. Under laboratory conditions, the biotrophic phase of this pathogen generally lasts from 9 to 14 days [23]. The biotrophic phase is followed by the necrotrophic (symptomatic) phase. Significant changes in the host’s (wheat’s) and the pathogen’s transcriptomes take place in the necrotrophic phase. During that stage, most changes in plant cells are apoptotic rather than necrotic [19]. The mechanisms of action of necrosis-inducing proteins (NEP1, ZtNIP1) produced by *Z*. *tritici* still remain poorly understood, but their presence points to interactions between necrotic factors and the defense response of wheat [19] (Table 1) (Figure 1).

In the necrotrophic phase, cell nutrients leak into apoplastic space, which significantly increases fungal biomass [21]. According to Cairns and Meyer (2017) [39], the secondary metabolites of *Z*. *tritici* with structural functions, such as melanin, a component of conidial cell walls, iron-chelating siderophores and substances that regulate the secretion of plant hormones, also play important roles during infection. The cited authors also demonstrated that *Z*. *tritici* relies on a different mechanism to accumulate and eliminate ferric ions than related fungal species, which can influence its infection potential. Moreover, most secondary metabolites of *Z*. *tritici* are produced by biosynthetic gene clusters composed of polyketide synthases (PKSs) or non-ribosomal peptide synthetases (NRPSs), and the neighboring genes encode tailoring enzymes and transporters [40].

Fungi of the genus *Fusarium*, including *F*. *graminearum* Schwabe (teleomorph: *Gibberella zeae*), *F*. *culmorum* (W. G. Sm.) Sacc. (no teleomorph described), *F*. *avenaceum* (Fr.) Sacc. (teleomorph: *G*. *avenaceae*) and *F*. *poae* (Peck) Wollenw. (no teleomorph known), cause *Fusarium* head blight (FHB) in wheat [41]. *Fusarium* fungi have similar infection cycles [42]. Six to 12 days after inoculation in the full flowering stage, macroconidia germinate on all host plants. Spore germination is influenced by temperature, spore distribution and the water potential of plant tissues. The relationship between temperature and ascospore discharge by *F. graminearum* has been investigated by several authors. Tschanz et al. (1976) [43] reported that the optimal temperature for spore germination ranges from 11 to 26 °C with a peak at 16.6 °C, and according to Schmale and Bergstom (2004) [44], the optimal temperature is 10–30 °C with a peak at 25 °C. The most recent data collected by Manstretta and Rossi (2016) [45] indicate that ascospore discharge is high between 15–25 °C and peaks at 21 °C. Those differences can be attributed to the influence of external factors. David et al. (2016) [46] demonstrated that the highest number of *F. graminearum* ascospores are released in cooler and more humid conditions; however, they travel the greatest distance in warmer and more humid conditions. There results should contribute to field management decisions made by farmers. Pathogenic *Fusarium* species can infect plants by colonizing: (1) the adaxial surfaces of glumes; (2) the lemmae; (3) the palea; and (4) wounds in the chaff [47]. Host tissues are not directly penetrated by hyphae which require 24–36 h to grow and colonize plant surfaces. Pathogens spread vertically from top to bottom, and they reach the rachillae, rachises and nodes. Hyphae penetrate vascular bundles and the cells surrounding vascular bundles. Pathogens produce enzymes cellulase, xylanase and pectinase which degrade plant cell walls and induce other changes in the infected cells, including deformation and degradation of the cytoplasm and cell organelles [48]. These enzymes are produced mainly in the first stage of infection [25,49]. The virulence of *F*. *graminearum* is determined by the expression of five main genes: *Tri5* which encodes the biosynthesis of deoxynivalenol, *Mgv1* and *Gpmk1* which encode protein kinases, *Cps1* which encodes the production of enzyme Cps1, and *Fgli1* which encodes the production of lipase virulence factors (Table 1). Mutants without the above pathogenicity genes were significantly less effective in colonizing wheat [28] (Table 1). In the initial stages of FHB, wheat spikes take on a light brown hue, turgor pressure inside spikes decreases, and spikes turn white. Necrotic changes gradually spread to the neighboring cells [50].

The fungal species *Parastagonospora nodorum* causes Septoria nodorum blotch in all wheat growing regions. Hyphae growing from germinating spores penetrate wheat tissues through both the cuticle and, opportunistically, through stomata. Previous studies conducted by Zinkernagel et al. (1998) [51] have demonstrated subcuticular growth of *P. nodorum*. The first stage of wheat tissue penetration is associated with swelling, both at the hyphal tip and on lateral branches, which partially resembles the fungal appressoria of *Z. tritici*. Pycnidia are produced after around 7 days, depending on humidity. These structures are produced throughout the lesion [52]. The primary site of leaf infection with *P. nodorum* turns yellow, and when leaf chlorosis is complete, the fungus spreads through the tissue, and asexual sporulation begins. Further spread of the infection leads to glume blotch [52]. Sexual ascospores are dispersed by wind, and asexual pycnidiospores are dispersed by rain splash. Genes are intensively exchanged between *P. nodorum* populations, which enriches the gene pool of the species [53], similarly to *Z. tritici* [54] and *Fusarium* spp. [29]. The analysis of the expression of *SNOG* genes encoding the synthesis of phosphate transporters in *P*. *nodorum*. Ipcho et al., (2012) [36] demonstrated that the uptake of inorganic phosphorus from host cells increases in early stages of infection to boost *P*. *nodorum* metabolism and to accumulate inorganic phosphorus in the form of polyphosphates. In the cited study, gene expression increased in the initial stages and decreased in successive stages of infection [36]. Genes encoding enzymes that degrade cell walls (xylanase, cellulase) and decompose proteins and carbohydrates in plant cells are also expressed in the early stages of infection [33]. The genes encoding the production of pathogenic ribosomes and genes responsible for nutrient assimilation and catabolic processes continue to be expressed until late stages of infection [55]. According to Pöggeler and Wöstemeyer (2011) [55], in the process of wheat infection with *P*. *nodorum*, pathogenicity effectors and cell wall-degrading enzymes are released into extracellular space to induce necrosis and disorganization of the neighboring cells and to produce simple metabolites that are later carried to fungal cells by protein transporters. One of the most important effector genes is *ToxA* which encodes protein with molecular mass of 13.2 kDa and induces tissue necrosis in *T*. *aestivum* cultivars that carry the *Tsn1* sensitivity gene [56] (Table 1). In both cases, the process ends with the loss of cellular fluid and tissue penetration by pathogenic fungi [57].

## 3. The Role of Morphological and Anatomical Barriers in Conditioning Resistance to Pathogens in Wheat

The anatomical and morphological traits of wheat are rarely studied despite the fact that they constitute the main mechanism of passive resistance to infection. Moreover, morphological features allow plants to escape disease. The anatomical barriers in wheat include the cuticle, waxy cuticle and trichomes (fine hairs). The cuticle covers the external walls of the epidermis, trichomes, stomatal pores and lacunae. It is composed mainly of cutin or cutan and wax. Cutin and cutan are polycarbonate polymers, and waxes are composed mainly of long-chain fatty acids (C20–C40) and their derivatives: alkanes, aldehydes, primary and secondary alcohols, ketones and esters [58]. The waxy layer is continuous, and it is not intersected by middle lamellae between adjacent cell walls. In wheat, the cuticle has a complex structure with layers of wax platelets and crystals. Wax structure is determined by environmental conditions, and it is negatively correlated with leaf hydrophobicity [59]. Water droplets on the surface of wheat leaves create a favorable environment for the germination of fungal spores. The mechanical properties of waxes can influence the adhesion and development of fungal spores. The thickness of waxy layers also determines plant resistance to pathogenic infections. The low-wax lines of wheat are generally characterized by higher yields and lower light-scattering effects, which enhances photosynthesis in the grain fill/ripening stage [60]. However, these properties also make low-wax wheat lines more susceptible to infections. Trichome structure plays an important role in wheat infections caused by *P. nodorum*. Wicki et al. (1999) [61] selected three varieties of winter wheat with the highest resistance to *P. nodorum* in field conditions. The three most resistant varieties had a strong wax layer on the ear [61]. It appears that the thick wax layer in wheat prevents *P. nodorum* from decomposing the cuticle. Waxes also play an important role in decreasing wheat’s susceptibility to FHB. According to Gunnaiah et al. (2012) [62], free fatty acids in the wax layer show higher fold change in wheat lines resistant to FHB than in susceptible lines.

Trichomes, specialized epidermal cells, are also an anatomical barrier which protects infected wheat against mechanical damage [63]. Trichomes exert a significant influence on potential *Z. tritici* infections despite the fact that this fungus infects plants primarily through stomata [17]. Fones et al. (2017) [64] demonstrated that trichomes on wheat leaves capture *Z. tritici* spores and improve their adhesion to these structures. Similar observations were made in *P. nodorum* pycnidia which were retained at the tip of trichomes, thus significantly reducing the number of spores bounded in leaf depressions between ridges [65].

Duncan and Howard (2000) [66] reported that the hyphae of germinating *Z*. *tritici* spores are directed towards open stomata which act as a gateway to infection. However, extensive microscopic research conducted by Shetty et al. (2003) [67] demonstrated that the majority of germ tubes grew in a direction opposite to the stomata, and hyphae growing in the direction of the stomata were observed only in selected cases. *Z. tritici* is also characterized by low expression of genes that encode cutinase and degrade cutin in early stages of infection in wheat, which is consistent with the model depicting the role of stomata in plant infections [68]. Stomata are also a gateway to *F. graminearum* [69] and *P. nodorum* infections [52]. *Fusarium graminearum* does not produce appressoria, but this fungus has been found to penetrate the adaxial surface and stomatal openings of glumes, lemma and palea [69].

## 4. Pathogen-Induced Resistance in Plants–Pattern-Triggered Immunity

During evolutionary processes, plants have developed sophisticated mechanisms of defense against pathogens. Active resistance is triggered by pathogens, and it involves biochemical and molecular responses. In pattern-triggered immunity (PTI), conserved virulence factors such as chitin, the main building block that makes up the cell wall in pathogenic fungi, are recognized by the host plant [70]. Pattern recognition receptors are able to recognize non-self structures referred to as pathogen-/microbe-associated molecular patterns or PAMPs/MAMPs [71]. The identification of PAMPs/MAMPs activates plant defense mechanisms such as the accumulation of reactive oxygen species and PR proteins, and it initiates lignification and callose deposition [72]. Shetty et al. (2009) [73] demonstrated that the PR2 protein (β-1,3-glucanase) in wheat is essential for cleaving *Z. tritici* cell walls and releasing elicitors that activate defense responses. Early accumulation of β-1,3-glucanase transcripts and chitinase was observed in resistant wheat lines [73]. Chitinase synthesis and accumulation supports quick identification of β-1,3-glucan, a component of *Z. tritici* cell walls, which inhibits the spread of this fungal pathogens in wheat tissues [73]. Chitin also elicits defense responses in wheat [74,75]. Fragments of *Z*. *tritici* chitin are recognized in the presence of chitin elicitor-binding protein (CEBiP) and chitin elicitor response kinase 1 (CERK1) (Figure 1) [76]. The wheat genome contains one gene encoding CEBiP and two genes encoding CERK1 [24]. Chitin elicitor-binding protein is a membrane protein that has been first identified in rice. It undergoes strong glycosylation during the interactions between the pathogen and the host plant. The characteristic features and the significance of CEBiP glycosylation in wheat and other cereals have not been elucidated. The lysine motif in the extracellular domain of CEBiP recognizes and binds chitin, but CEBiP does not have an intracellular signaling domain, and it is unable to initiate an immune signal (Figure 1). The CERK1 transmembrane protein comprises an extracellular domain with a lysine motif and an intracellular kinase domain, and it is capable of inducing a signal transduction cascade [76]. Mitogens (MAPK) activate kinases, induce the expression of *WRKY* (*WRKY53* and *WRKY33*) genes, and initiate the signal transduction cascade (Figure 1). WRKY genes encode WRKY proteins which act as transcription factors during a pathogenic infection [77]. In *Z*. *tritici*, the expression of the *Mg3LysM*-encoding gene is intensified only in the asymptomatic phase. The expression of the *Mg3LysM* gene decreases in the necrotic phase, which indicates that the produced protein participates in chitin sequestration only in the first stage of infection (Figure 1) [24]. Interestingly, Lee at al. (2014) [24] observed that the Z. *tritici* mutant without a functional *Mg3LysM* gene, which normally decreases virulence, was highly virulent in wheats where CEBiP and CERK1 proteins had been silenced by the virus-induced gene silencing (VIGS) method. This indicates that virus silencing of CEBiP and CERK1 receptors allows Mg3LysM, normally a nonpathogenic deletion mutant in *Z. tritici*, to colonize the leaf. It also underlines the importance of these receptors in chitin-induced defense. 

Pattern-triggered immunity to *Fusarium* pathogens has been widely investigated in wheat cultivar Sumai 3 [78]. When a *Fusarium* infection is signaled, wheat synthesizes two hydrolase enzymes, β-1,3-glucanase and chitinase [79], which is consistent with wheat’s defense responses to *Z. tritici* infections [73]. Chitin, the building block of the cell wall in fungal pathogens, elicits a defense response in infected plants [80]. β-1,3-glucan and chitin are the main components of fungal cell walls, and their recognition initiates the inhibition of fungal growth in plant tissues [81]. The enzymatic activity of β-1,3-glucanase and chitinase is higher in more resistant cultivars (such as Sumai 3) than in cultivars that are more susceptible to *Fusarium* infections (such as Xiaoyan) [78]. Higher resistance to *Fusarium* infections leads to the accumulation of phenolic compounds, proteins and glycoproteins rich in hydroxyproline in cell walls [49]. Wheat also responds to pathogenic infections by accumulating callose in infected tissues [78]. To date, host defense mechanisms associated with resistance to deoxynivalenol and other group B trichothecenes have been described at the molecular level, because these compounds induce necrotrophic changes and facilitate colonization by *Fusarium* fungi [48].

Plant immunity is influenced by: (1) the ability to glycosylate mycotoxins [82]; (2) mycotoxin tolerance [83]; and (3) agronomic treatments and crop protection agents [84]. In wheat lines resistant to type 1 FHB (Fhb1), glycosylation leads to the accumulation of DON-3-*O*-glucoside (D3G), which indicates that DON is decomposed during biochemical processes [85]. In *Arabidopsis thaliania*, the UDP-glucosyltransferase (UGT) enzyme detoxified DON by converting it to D3G, which suggests that UGT enzymes and their regulators are encoded by the *Fhb1* gene [86].

## 5. Gene Expression in Infected Wheat

Wheat infections caused by *Z*. *tritici* decrease the expression of genes responsible for defense responses in host plants [21], mainly in the asymptomatic phase. According to estimates, the expression of up to 60% of genes which are involved in defense against *Z*. *tritici* decreases as a result of pathogen recognition by the plant. The expression of pathogenesis-related (PR) genes–*PR1*, *PR2* (encoding glucan endo-1,3-β-glucosidases) and PR3 (encoding basic chitinases)—is compromised in wheat infected with *Z*. *tritici* [21,73]. Changes in the concentrations of calcium ions, calmodulin- and calcium-transporting ATPase proteins, which are important sensors and mediators of Ca^2+^-dependent signals, are also observed on the first day of infection with *Z*. *tritici*. Similar to PR genes, the expression of genes encoding the above molecules is decreased [18,87]. The expression of genes encoding proteins that detoxify xenobiotics: enzymes of the glutathione transferase family, cytochrome P450s, ATP-binding cassette transporter and UDP glucosyl/glucuronyl transferases is also inhibited as a result of wheat-*Z. tritici* interactions. The activity of the above genes increases gradually during the infection and is much higher during the transition from the asymptomatic phase to the necrotic phase. A similar increase is observed in the expression of the lipoxygenase 2 gene, whose product is the first element of the jasmonic acid biosynthesis pathway, as well as genes encoding the synthesis of fructan-metabolizing enzymes which are a valuable source of energy in wheat leaves exposed to biotic stress [87]. In the discussed stage, wheat begins to accumulate substances for the synthesis of defense-related compounds and proteins. The expression profile of genes involved in wheat responses to *Z. tritici* infections was analyzed by Yang et al. (2013) [18] who observed changes in the expression of host transcripts involved in the host’s metabolism (in particular carbohydrate metabolism), transport, signaling and defense mechanisms with substantial up-regulation of transcripts during the necrotic stage. The cited author also demonstrated that the genes encoding photosynthesis were down-regulated in that stage. The transcripts involved in anti-oxidative stress were also down-regulated at the end of necrotrophic stage, which was correlated with the high accumulation of H_2_O_2_ [18]. Previous research demonstrated that H_2_O_2_ plays a key role in wheat defense during the biotrophic phase of wheat-pathogen interactions. Surprisingly, unlike strictly necrotrophic pathogens, the hemibiotrophic *Z. tritici* does not benefit from the presence of H_2_O_2_ in the necrotrophic phase. H_2_O_2_ is considered to be harmful to *Z. tritici*; however, it can be tolerated [67,88]. The toxicity of H_2_O_2_ for the pathogen depends on its concentration. In an in vitro study, Shetty et al. (2007) [88] demonstrated that at the optimal concentration, H_2_O_2_ effectively inhibited the development of *Z. tritici* inoculum. The role of H_2_O_2_ and other reactive oxygen species in pathogen-plant interactions has been described in detail by Shetty et al. (2008) [89]. *Z. tritici* interactions have also demonstrated that the wheat protein disulfide isomerase gene is expressed in infected wheat cells [90] and that structural defense responses take place [73]. Biochemical analyses revealed DNA laddering, cytochrome c translocation (from mitochondria to the cytoplasm), loss of host cell membrane integrity, degradation of total wheat RNA and differential regulation of host mitogen-activated protein kinase (MAPK) pathways [91,92]. Studies into wheat responses to *Z. tritici* infections revealed that the pathogen penetrates wheat leaves by suppressing host defense responses during the asymptomatic biotrophic stage, activating signaling mechanisms and acquiring apoplastic nutrients. However, necrotrophic growth enhances defense mechanisms in the host organism, such as energy metabolism, signaling and transport, and it decreases the rate of photosynthesis [18].

**Figure 2:**
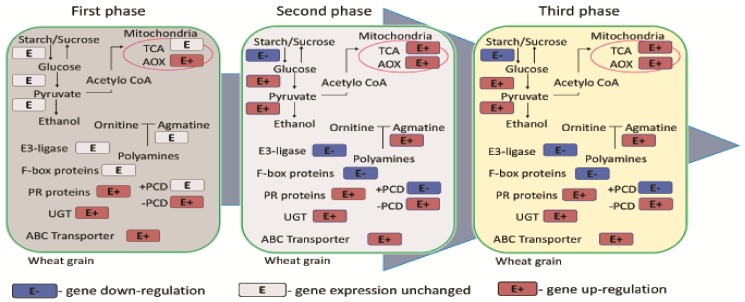
Metabolic pathway of the three main stages of infection caused by *F. graminearum* in wheat (adapted from Chetouhi et al. (2016) [50]). AOX: alternative oxidase, E: gene expression unchanged; E+: gene up-regulation; E−: gene down-regulation; PCD: programmed cell death; PR: pathogenesis-related genes; TCA: tricarboxylic acid; UGT: UDP-glucuronosyltransferase. Modifications: emphasis on gene expression profiles using “+” and “−“ indicators, simplification of graphic presentation.

Wheat infections caused by fungi of the genus *Fusarium* gradually decrease the expression of genes encoding the synthesis of starch and sucrose in grain cells. This process benefits pathogens. During glycolysis, glucose accumulated in grain is converted to simple components which are a source of energy for fungal pathogens and promote the spread of the infection in wheat spikes [86]. The three main phases of *F. graminearum* infection in wheat are presented in Figure 2. During the first phase, hyphae gradually grow into plant tissue, and this stage is accompanied by the synthesis of DON [50]. Mycotoxins synthesized by *Fusarium* spp. fungi probably inhibit programmed cell death (PCD) by decreasing the expression of genes encoding this process. The above increases the expression of genes encoding the alternative oxidase (AOX) enzyme in plants. Alternative oxidase prolongs the life of plant cells and protects them against mycotoxins. In the second phase of infection, *F. graminearum* proliferates inside wheat tissues, and DON synthesis is inhibited ([50], Figure 2). Symptoms of wheat infection with *Fusarium* spp. are manifested, and the plant initiates DON neutralization mechanisms [93]. Pathogenic infections also inhibit the expression of genes encoding F-box subunits of the E3 ubiquitin ligase complex. These genes are responsible for PCD, and they participate in the immune responses of infected plants [50] (Figure 2). In naturally infected plants, the expression of the genes encoding UGT increases in successive stages of disease. Recent research has focused on the overexpression of UGT in the wheat genome which increases resistance to FHB [94,95]. The third phase of infection marks the second spurt of fungal growth without mycotoxin synthesis (Figure 2). This phase involves changes in carbohydrate metabolism, in particular the expression of genes encoding the tricarboxylic acid cycle [50].

The molecular interactions between *P. nodorum* and wheat have been described mainly in wheat lines carrying specific sensitivity loci to *P. nodorum* necrosis effectors [96,97,98]. The pathogen produces necrotrophic effectors (previously described as host-specific toxins SnToxN encoded by *SnToxN* genes) that interact with wheat susceptibility genes. The severity of the disease depends on the number and identity of effectors and wheat susceptibility genes [99]. SnTox1 (10–30 kDa) interacts with the *Snn1* gene present on wheat chromosome arm 1BS [100]. It is believed that this interaction accounts for around 60% of variation in disease. In wheat lines harboring the *Snn1* gene, necrosis is induced in the presence of the SnTox1 toxin [100]. After SnTox1 recognition, wheat initiates a classic defense response by increasing the expression of the *PR1-1-1* gene, the thaumatin-like protein gene and genes encoding chitinase synthesis [101]. The second type of interactions between the toxin and the sensitivity gene is SnToxA-*Tsn1*. Sensitivity to this toxin is inherited dominantly, and it is dependent on *Tsn1* (localized on the long arm of chromosome 5B in wheat) [97]. Molecular cloning of *Tsn1* revealed a 1490 amino-acid protein consisting of resistance gene-like serine/threonine protein kinase, nucleotide binding and leucine-rich repeat domains. All amino acid proteins are required for ToxA sensitivity in wheat [97]. According to research, protein Tsn1 is not a receptor for ToxA because the *Tsn1* gene does not encode integrin-like protein. Tsn1 protein can act as a guard for the cellular membrane-spanning ToxA receptor [97]. Tsn1 expression is regulated by the circadian clock, and toxin-susceptibility interactions are considered to be light dependent [102]. These interactions disrupt the photosystem and lead to cell death [98]. Other examples of toxin-sensitivity gene interactions include SnTox2-*Snn2* [103], SnTox3-*Snn3* [104] and SnTox4-*Snn4* [99]. The *Snn2* sensitivity gene is localized on chromosome 2D [103], *Snn3* genes–on the short arms of chromosomes 5B and 5D [104], and the *Snn4* gene–on the short arm of chromosome 1A [99]. The interactions between SnTox2-*Snn2* and SnTox4-*Snn4* are light dependent, whereas no such correlations were observed in the two remaining genes.

## 6. Characteristics of Loci in the Wheat Genome Conferring Resistance to Pathogenic Infections

At least 20 loci (*Stb* loci) encoding qualitative resistance to *Z*. *tritici*, often isolate-specific resistance, have been identified in the wheat genome. In hexaploid wheat, resistance is conditioned mainly by *Stb1*, *Stb18*, *StbSm3* and *StbWW* loci (Table 2). However, these loci determine resistance to only a small group of *Z*. *tritici* isolates [105]. Many wheat cultivars grown in Europe, China, Israel and the United States, harbor the *Stb6* gene which is responsible for partial resistance to *Z*. *tritici* in British wheat (Table 2). Another promising gene is *Stb16q* which effectively conditioned resistance to all tested isolates of *Z*. *tritici* in wheat [106] (Table 2). Wheat genotypes TE91111, Kavkaz-K4500 L.6.A.4 and Veranopolis have been used as sources of resistance against STB, and each of them harbors more than three genes conditioning resistance to the disease [107]. The above suggests that the accumulation of resistance gene could be an effective strategy for breeding highly resistant wheat cultivars [19]. A high number of QTLs determining resistance to *Z*. *tritici* have also been mapped. Many of them were localized in the vicinity of genes encoding qualitative resistance. However, the identified QTLs were less effective in conditioning resistance in wheat than the neighboring single genes [106].

Host plants develop quantitative resistance to infections caused by *Fusarium* fungi, which implies that a large number of genes are involved in the process of encoding a given trait. Some of the analyzed QTLs are characterized by a Mendelian pattern of inheritance [113]. In the model common wheat cv. Sumai 3, a short arm of chromosome 3B carries the *Fhb1* gene, the main QTL conditioning resistance to FHB (locus Qfhs.ndsu-3BS). Type 1 resistance to infections is also conditioned by a gene on chromosome 5A (locus Qfhs.ifa-5A), whereas the *Fhb2* gene conditioning type 2 resistance to *Fusarium* pathogens colonizing wheat spikes has been localized on chromosome 6B [113] (Table 2). The resistance genes in wheat cv. Sumai 3 prevent the germination of fungal spores and inhibit the spread of the infection in the host plant’s tissues. A large number of QTLs conditioning resistance to FHB have been described by Buerstmayr et al., (2009) [116]. In addition to *Fhb1* and *Fhb2*, which are the main sources of resistance, resistance to FHB is also conditioned by Fhb3 in Leymus racemosus, *Fhb4* and *Fhb5* in *T. aestivum* cv. Wangshuibai, and *Fhb6* in *Elysmus tsukushiensis* [114]. In recent years, new QTLs encoding resistance to FHB have also been identified in plant species related to wheat, including *Thinopyrum ponticum* (Podp.) Barkworth & D.R. Dewey (2n = 10× = 70) and *E*. *repens* [114,117].

In wheat, resistance to Septoria nodorum blotch is controlled by several loci that are inherited independently. Genes at these loci are exposed to environmental factors and pleiotropic effects (in particular relating to plant growth and the heading stage) [61]. Similar to FHB, resistance to Septoria nodorum blotch is conditioned mainly quantitatively, but monogenic resistance has been observed in common wheat [118] and *Triticum tauschii* (Coss.) Schmalh. [119], whereas in *Triticum durum* Desf., it was inherited from *Triticum timopheevi* (Zhuk.) Zhuk. [120]. The main QTL conditioning resistance to Septoria nodorum blotch is *QSng*.*sfr*-*3BS* which was responsible for more than 30% of phenotypic variation in recombinant inbred lines (RILs) of hybrids of common wheat cultivars Arina and Forno (Swiss cultivars of winter wheat). The *QSng*.*sfr*-*3BS* gene is located at the terminus of the short arm of wheat chromosome 3B [76] (Table 2). Tommasini et al., (2007) [121] demonstrated that this QTL was also responsible for more than 40% of genetic variation in 44 European cultivars of winter wheat. The cited authors developed a genetic map confirming the conserved character of *QSng*.*sfr*-*3BS* in modern cultivars of winter wheat, which paves the way for future research into increasing the frequency of desirable alleles in the gene pool of wheat. *Qsnb*.*fcu*-*1A* on wheat chromosome 1A is also an important QTL which has been identified in hybrids of wheat cultivars Arina and Forno [99,121] (Table 2). Adhikari et al., (2011) [108] relied on the association mapping (AM) technique and Diversity Arrays Technology (DArT) markers to confirm the presence of 24 DArT markers conditioning resistance to *P. nodorum* on wheat chromosomes 1A, 2D, 3B, 5B and 7A. Chromosomes 2D, 3B and 5B correspond to the previously identified QTLs responsible for resistance to Septoria nodorum blotch [122,123], and the remaining chromosomes are a new source of resistance that can be used in breeding for disease resistance in wheat.

## 7. Practical Application Wheat Defense Mechanisms

Agronomic treatments and plant protection agents only partially counteract the growth of pathogens, which is why resistance breeding and improvement of plant defense mechanisms are the key wheat protection strategies. Research and breeding efforts aiming to improve wheat resistance to pathogens follow two main avenues: (1) introduction of effective resistance genes through translocation; and (2) increasing inherited resistance traits such as quantitative traits [124]. Research into transgenic plants resistant to FHB focuses on the overexpression of genes encoding proteins which participate in plant defense responses and genes encoding cell wall proteins which inhibit the spread of infection [125,126,127,128]. Crop protection products such as cell wall fragments, plant extracts and synthetic compounds can enhance resistance at both local and systemic levels [129]. Integrated plant protection methods rely on systemic acquired resistance (SAR) which is induced by molecules synthesized by the pathogen as well as molecules produced by the infected plant [130]. When identified, isolated and industrially synthesized, these molecules can be used to develop protection products that artificially induce resistance in plants. Makandar et al., (2012) [125] demonstrated that salicylic acid also elicits resistance in wheat and can be effectively used to protect wheat against FHB. Salicylic acid plays a similar role in wheat resistance to *Z*. *tritici* [131]. Cuperlovic-Culf et al., (2016) [132] conducted a metabolomic study into substances that stimulate plant defenses against FHB, including spermine and putrescine. Motallebi et al., (2017) [133] used methyl jasmonate (MeJA) to protect durum wheat inoculated with *F*. *culmorum*, which increased the expression of genes encoding immune responses to infection: phenylalanine ammonia-lyase (PAL), lipoxygenase (LOX), cytochrome P450 (CYP709C1) and selected PR genes (*PR3*, *PR4* and *PR9*). According to Lewandowski et al., (2014) [134], research efforts are under way to develop crop protection products containing various immunity inducers and modified molecules of the existing SAR inducers.

As mentioned earlier, the gene pool of modern wheat varieties continues to decrease [4]. New sources of allelic variation, such as relict wheat species, should be identified to preserve biodiversity [135]. However, the deployment of these alleles in wheat breeding is influenced by the distribution of genetic diversity across genes that encode particular traits and the recombination rate near these genes which can be potentially suppressed. For this reason, new molecular techniques supporting direct manipulation of plant genes appear to be highly promising. The CRISPR/Cas system for genome editing has attracted considerable interest in recent years [136]. CRISPR/Cas is regarded as a useful tool for overcoming the limitations associated with the decrease in allelic variation [136]. The CRISPR/Cas system is composed of clustered, regularly interspaced, short palindromic repeats associated with protein genes (Cas). The system is a part of the adaptive immune system in bacteria and archaea. It can recognize specific DNA sequences and degrade them when they reappear in the organism [137]. CRISPR/Cas has been applied to modify the genomes of numerous crops and model plants, including tobacco [138], barley [139] and wheat [140,141,142]. Continued progress in genome editing systems will accelerate the development of new wheat varieties with novel variations in genomic regions that control, for example, resistance traits. Several strategies of multiplex gene editing in plants have been proposed to date [143,144,145,146]. However, the progress that has been made in the CRISPR/Cas system indicates that molecular components need to be optimized for large-scale applications.

Other techniques that rely on RNA to mediate protection against fungal infections include host-induced gene silencing (HIGS) and spray-induced gene silencing (SIGS). Target-specific inhibitory RNA is regarded as an alternative fungicide treatment for conventional cereals [147]. At present, cereals are protected against FHB with demethylation inhibitor (DMI) fungicides which inhibit ergosterol biosynthesis by binding to cytochrome P450 lanosterol C-14α-demethylase (CYP51) [148]. Koch et al. (2013) [149] investigated the potential of HIGS targeting the *CYP51* genes (necessary for ergosterol biosynthesis) in fighting *F. graminearum* infections. Their experiment revealed that silencing of *CYP51* inhibits the pathogen’s growth. It also demonstrated that HIGS of these genes is an effective strategy for controlling the diseases caused by *Fusarium* [149]. However, many scientists have questioned the broad applicability of HIGS due to GMO restrictions and food production strategies in many countries. Koch et al. (2016) [150] relied on SIGS to apply *CYP3*-dsRNA (targeting three fungal ergosterol biosynthesis genes: *CYP51A*, *CYP51B* and *CYP51C*) and target *F. graminearum*. Spray applications also effectively inhibited the growth of *F. graminearum*. The use of gene silencing techniques in plants also requires the optimization of factors that contribute to the success of RNA interference. These factors include the structure of double-stranded RNA, its length, combinatorial order of gene fragments, target sites and the number of potential genes targeted by one dsRNA [147].

## 8. Conclusions

The pathogens described in this review pose the greatest threat to wheat in Central Europe and in countries with similar geoclimatic conditions, high humidity and low average temperatures during the growing season. *Z. tritici* infects wheat seedlings and leaves, *P. nodorum* infects wheat plants in the heading stage, and *Fusarium* fungi colonize wheat seedlings and wheat plants during stem elongation, flowering and ripening. These pathogens are present in farms nearly every year. Plant resistance mechanisms against pathogens, both at the level of anatomical and morphological structures and at the genetic level, attract the growing interest of researchers and breeders. The analyzed pathogens have completely different mechanisms of action, and new information about these processes is provided by molecular studies. The defense mechanisms of wheat against fungi of the genus *Fusarium* and *P. nodorum* have been most widely researched. The discussed pathogens produce molecules that increase their virulence. Fungi of the genus *Fusarium* synthesize numerous mycotoxins, whereas *P. nodorum* produces toxic necrotrophic effectors. *Z. tritici* suppresses immune responses in host plants, and this unique mechanism of action is not encountered in infections caused by fungi of the genus *Fusarium* or *P. nodorum*. The search for genes conditioning resistance to *Z. tritici*, a pathogen that causes massive crop losses around the world, poses a new challenge. Research into the genetic determinants of resistance in selected wheat cultivars will contribute to resistance breeding by introducing resistance genes into new breeding material. The interactions between the pathogen and the host plant have to be well understood to develop novel methods for protecting wheat against pathogens, including new varieties and genetically modified varieties.

## Figures and Tables

**Table 1:** Major genes controlling the infection cycle of *Z. tritici*, *F. graminearum* and *P. nodorum*.

Pathogen	Gene	Gene Expression Interaction Stage	Encoded Trait	Author
***Z. tritici***	*Mg3LysM*	Colonization	Suppression of defense responses in wheat in the first stage of infection	[23,24]
	*NEP1*	Formation of fruiting bodies	Necrotic factor	[19,23]
	*ZtNIP1*	Formation of fruiting bodies	Necrotic factor	[19,23]
	*Cellulase genes*	Formation of fruiting bodies	Production of cellulase, a cell-wall degrading enzyme	[23,25]
	*Xylanase genes*	Formation of fruiting bodies	Production of xylanase, a cell-wall degrading enzyme	[23,25]
	*Pectinase genes*	Formation of fruiting bodies	Production of pectinase, a cell-wall degrading enzyme	[23,25]
***F. graminearum***	*Tri5*	Colonization	Deoxynivalenol (DON) synthesis	[26,27]
	*Mgv1*	Sexual reproduction	Fecundity, production of heterokaryons; formation of fungal cells; virulence	[28,29]
	*Gpmk1*	Sexual reproduction	Formation of ascospores and perithecia; virulence	[28,29]
	*Cps1*	No data	Production of enzyme CPS1 composed of two AMP-binding domains with an unknown biochemical faction, a potential virulence factor	[30]
	*Fgl1*	Colonization	Production of lipase, a potential virulence factor	[31,32]
	*Cellulase genes*	Colonization/Penetration	Production of cellulase, a cell-wall degrading enzyme	[31,33]
	*Xylanase genes*	Colonization/Penetration	Production of xylanase, a cell-wall degrading enzyme	[31,33]
***P. nodorum***	*ToxA*	Colonization	Necrotic factor	[34,35]
	*SNOG*	Colonization	Family of genes encoding the synthesis of various phosphate transporters	[36]
	*5S ribosomal*	During all stages of infection	Synthesis of 5S ribosomal subunits	[37]
	*Cellulase genes*	Colonization/Penetration	Production of cellulase, a cell-wall degrading enzyme	[36,38]
	*Xylanase genes*	Colonization/Penetration	Production of xylanase, a cell-wall degrading enzyme	[36,38]

**Table 2:** Genes encoding resistance to selected pathogens in wheat.

Pathogen	Resistance Gene	Chromosome	Author
***Z. tritici***	*Stb1*	5BL	[108]
	*Stb18*	6DS	[109]
	*StbSm3*	3AS	[106]
	*StbWW*	1BS	[110]
	*Stb6*	3AS	[111]
	*Stb16q*	3DL	[112]
***F. graminearum***	*Fhb1*	3BS, 5AS	[113]
	*Fhb2*	6BS	[113]
	*Fhb3*	7AL	[114]
	*Fhb4*	4BL	[114]
	*Fhb5*	5AS	[114]
	*Fhb6*	1AS	[114]
***P. nodorum***	*Qsng.sfr.3BS*	3BS	[115]
	*Qsnb.fcu-1A*	1A	[99]

## Abbreviations

AM	Association mapping
AOX	Alternative oxidase
Cas	CRISPR associated
CEBIP	Chitin elicitor-binding protein
CERK1	Chitin elicitor response kinase 1
CRISPR	Clustered Regularly-Interspaced Short Palindromic Repeats
DArT	Diversity Arrays Technology
DMI	Demethylation inhibitors
DON	Deoxynivalenol
ETI	Effector-triggered immunity
FHB	*Fusarium* head blight
HIGS	Host-induced gene silencing
HR	Hypersensitive response
LOX	Lipoxygenase
LysM	Lysine motif
MAPK	Mitogen-activated protein kinase
MeJA	Methyl-jasmonate
NEP1	Necrosis and ethylene-inducing peptide
NIP	Necrosis-inducing proteins
NRPSs	Nonribosomal peptide synthetases
PAL	Phenylalanine ammonia-lyase
PCD	Programed cell death
PKSs	Polyketide synthases
PR	Pathogenesis-related
PTI	Pattern-triggered immunity
QoI	Quinone outside inhibitors
QTL	Quantitative trait loci
RILs	Recombinant inbred lines
ROS	Reactive oxygen species
SA	Salicylic acid
SIGS	Spray-induced gene silencing
STB	Septoria leaf blotch
TCA	Tricarboxylic acid
TGS	Transcriptional gene silencing
UGT	UDP-glucuronosyltransferase
VIGS	Virus-induced gene silencing
